# Acquired hemophilia A following COVID-19 vaccine: a case report

**DOI:** 10.1186/s13256-023-03850-z

**Published:** 2023-03-27

**Authors:** Bouselama Emna, Zahra Kmira, Ben Ismail Hajer, Sassi Nadia, Dhaha Yossra, Bouatay Amina, Ben Youssef Yosra, Regaieg Haifa, Khelif Abderrahim

**Affiliations:** 1 Department of Clinical Hematology, Farhat Hached University Hospital, Sousse, Tunisia; 2 Laboratory of Hematology, Sahloul University Hospital, Sousse, Tunisia

**Keywords:** Vaccines, COVID-19, Acquired Hemophilia A

## Abstract

**Background:**

In the literature, reported cases of Acquired hemophilia A (AHA) induced by COVID-19 vaccination occurred after Adenoviral Vector Deoxyribonucleic Acid (DNA)- and SARS-CoV-2 Messenger Ribonucleic acid (mRNA)-Based vaccines. Here, and to the best of our knowledge, we report the first case of AHA occurring after an inactivated Sinovac-coronavac COVID-19 vaccine.

**Case presentation:**

A 69-year-old Tunisian male patient consulted for severe left leg pain limiting physical mobility due to a 5*6 cm large ecchymosis located at the left inner thigh, having spontaneously appeared 5 days prior consultation and without notion of trauma. The patient had no known personal medical history. He had received the second dose of CoronaVac-SinoVac vaccine 30 days prior to consultation. Further physical examination revealed the presence of two other ecchymoses: one at the inner face of the right forearm, starting at the wrist reaching the elbow and the other at the left flank of the abdomen. Diagnosis of AHA was based on clinical presentation and confirmed with prolonged a PTT, Factor VIII deficiency and the presence of an FVIII inhibitor. The patient was successfully treated with corticosteroids and low dose Rituximab.

**Conclusion:**

Clinicians should consider AHA in front of prolonged aPTT with or without spontaneous bleedings even after inactivated virus COVID-19.

## Introduction

In December 2019, a global health crisis arose due to the highly contagious severe acute respiratory syndrome coronavirus 2 (SARS-CoV-2). Up-to-date, there is no effective drug for treating COVID-19 despite many trials [1]. This resulted in an unprecedented race by pharmaceutical companies attempting to develop effective vaccines. Shortly after initiation of COVID-19 vaccination, a number of serious vaccine-related diseases such as myocarditis [2], vaccine-induced immune thrombotic thrombocytopenia [3], and acquired hemophilia A (AHA) [4–7] were reported. As opposed to congenital hemophilia, AHA is a rare autoimmune disease due to the production of IgG autoantibodies to coagulation FVIII that burdens high morbidity and mortality [8]. The exact pathophysiology behind AHA remains uncertain, with a probable genetic predisposition, an association to certain underlying diseases (autoimmune disorders, respiratory diseases, allergic reactions, malignancies or hematologic malignancies) or to a triggering factor such as infections or pregnancies [8, 9]. The diagnosis is difficult in front of the lack of history of bleeding. It is presumed mainly on identifying an increased activated partial thromboplastin time (aPTT), even without bleeding and is confirmed by determining a FVIII inhibitor [9]. In this article we report a case of AHA occurring after an inactivated sinovac-coronavac COVID-19 vaccine.

## Case report

A 69-year-old Tunisian male patient consulted the emergency department at the month of June, for severe left leg pain limiting physical mobility due to a 5*6 cm large ecchymosis located at the left inner thigh, having spontaneously appeared 5 days prior consultation and without notion of trauma.

The patient had no known personal medical history, a family history of gastric cancer (father) and no psycho-social relevant history. He had received the second dose of CoronaVac-SinoVac vaccine 30 days prior to consultation. The patient reported a small ecchymosis appearing and disappearing spontaneously 3 weeks after the vaccination.

**Fig. 1:**
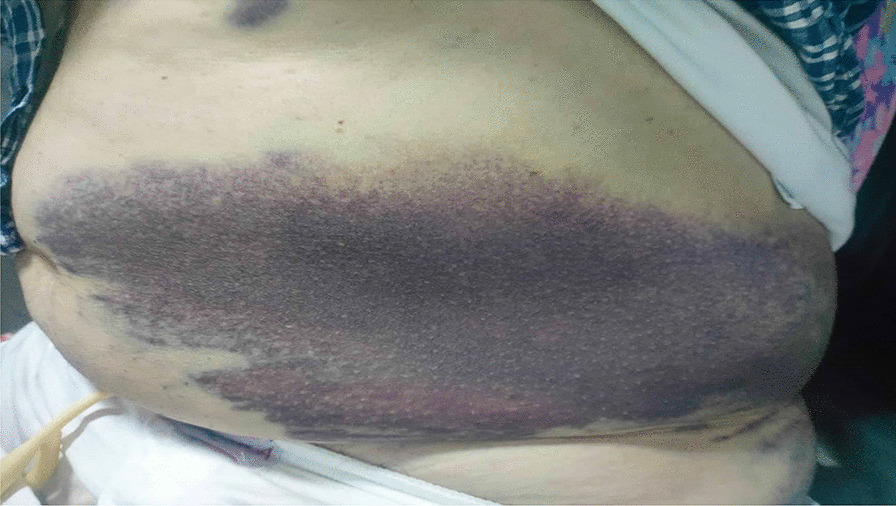
Ecchymotic spot on the left flank of the abdomen reaching the left iliac crest

Further physical examination at the the emergency department (ED) revealed the presence of two other ecchymoses: one at the inner face of the right forearm, starting at the wrist reaching the elbow, measuring 15 * 5 cm and the other at the left flank of the abdomen reaching the left iliac crest, measuring 40 * 12 cm (Fig. 1).

Both of these were secondarily reported by the patient as having spontaneously started 5 days ago, with no context of trauma. Soft tissue ultrasound performed in regard of the initial ecchymosis at the left inner thigh because of a 5 cm-asymmetry between the two thigh sizes revealed a hyper echoic infiltration of soft tissue located at the left inner tight.

The complete blood count showed marcrocytic normochromic anemia with hemoglobin at 8.4 g/dl; mean corpuscular volume at 103 fl; mean corpuscular hemoglobin at 32 pg/cell, hyper-reticulocytosis at 130 000/mm^3^ and a normal platelet count at 475 10^9^/l.

The determination of Prothrombin Time (PT), of aPTT and of coagulation factors were performed by coagulometric technique on ACL TOP. In our patient’s case, coagulation tests showed an isolated prolonged a PTT (100 s) which was not corrected on the mixing study.

In front of the presumptive diagnosis of acquired hemophilia A (AHA), treatment by systemic corticotherapy of 80 mg/die of prednisolone for 3 days was immediately initiated and switched by oral corticotherapy at the dose of 1 mg/kg. The patient received also tranexamic acid 3 g/die and red blood transfusion to correct the anemia. Bypass therapy was not considered if front of the absence of life-threatening bleeding.

In front of the patient’s age, the absence of history of bleeding or heparin-based treatment and the spontaneous occurrence of hematomas and ecchymoses associated with the isolated prolonged a PTT, specific determination of coagulation factors was conducted (available 13 days later), revealing an isolated decrease of factor VIII (FVIII) activity (F VIIIc equal to 1%).

FVIII deficiency was confirmed on two separate samples. The presence of an FVIII inhibitor was confirmed and titrated by the modified-Bethesada assay (BA) = 121 Bethesada units/ml, affirming the diagnosis of AHA.

The diagnosis of AHA was confirmed. After 17 days of corticosteroid treatment, the patient continued to show minimal improvement although the progressive shortness of aTTP and a course of rituximab 375 mg/m^2^ weekly for four consecutive weeks was initiated.

To exclude possible secondary organic causes of hemophilia A, CT-total body was performed with no signs of malignancy. Screening for autoimmune (anti-nuclear antibody, anti-extractable nuclear antigen, anti-double-stranded DNA), lupus anticoagulant studies, and chronic infectious diseases also resulted negative. There was also no history of illicit drug intake or recent medication.

**Fig. 2:**
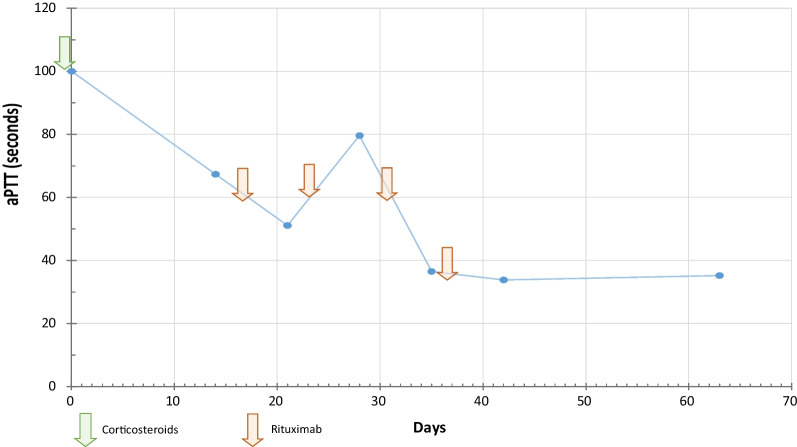
Timeline treatment/aPTT

The patient was later discharged after the first dose of rituximab and followed-up weekly at the out-patient clinic. After the second dose of rituximab, we noticed a prolongation of a TTP without worsening of the hemorrhagic syndrome not leading us to the interruption of treatment given its delayed effect. Follow-up showed a significant clinical (healing of the ecchymoses and no reoccurrence of further signs of bleeding) and normalization of a PTT 18 days of treatment with corticosteroids associated with rituximab. The detailed therapeutic schedule along with the biological improvement trends over 63 days are displayed in Fig. 2. There were no treatment-related adverse effects.

## Discussion

AHA is a rare autoimmune disorder resulting from the production of autoantibodies against FVIII affecting mainly the elderly with an incidence of around 1.4 cases per 1.000.000 [10] and leading to high morbidity and mortality especially if untreated. AHA should be suspected in front of an isolated prolonged a PTT with or without bleeding and confirmed by detecting a FVIII inhibitor [9]. Mostly with no identifiably trigger (43.6% to 51.9% of cases) [9], AHA has been described to be associated with genetic predispositions, certain underlying diseases (such as autoimmune disorders, respiratory diseases and malignancies) and infections or pregnancies [8, 9].

Up-to-date, there is no effective drug for treating COVID-19 despite many trials [1]. This resulted in an unprecedented race by pharmaceutical companies attempting to develop effective vaccines [11]. This promptitude along with the emergency use authorization accorded to many vaccine led people to question vaccination safety and policy [12]. In the literature, there have been many cases of AHA occurring after COVID-19 vaccination [4, 7]. Evidence about the causality of COVID-19 vaccines in the occurrence of a particular auto-immune phenomena thus causing autoimmune still remains unclear and debated [13].

The strength of this study is that, to the best of our knowledge, it reports the first occurrence of AHA after an inactivated COVID-19 vaccination (Sinovac-CoronaVac). In this case, it occurred 30 days after the second dose of the Sinovac-CoronaVac vaccine. Although it is very difficult to certainly conclude the causal relationship between the vaccines and AHA, and that the development of FVIII inhibitors post vaccination could be only a coincidental event, we believe that the association is valid in the reported case following a bundle of anamnestic, clinical, biological data as well as the timing between symptom-onset and vaccination. This is supported by absence of the common causes of AHA in our patient who did not have history of previous bleeding with negative autoimmune screen and no malignancies.

This study has some limitations. First, it reports the case of only one patient. Second, although the probable causal relationship between the AHA and the vaccine, eliminating a prior SARS-CoV-2 infection was not possible retrospectively.

Currently, no post-vaccination surveillance programs recommends screening for clinical or biological signs of AHA, nor any other of the rare autoimmune disorders potentially linked to COVID-19 vaccines. Attention needs to be paid in the post-vaccine period mainly based on auto-surveillance. Apart from presentations with spontaneous or uncontrolled bleeding, clinicians must consider the diagnosis of AHA when faced with isolated prolonged a PTT. Treatment is focused on symptomatically treating any bleeding along with etiological treatment. In urgent situations such as uncontrolled bleeding, the administration of recombinant FVII activated or the activated prothrombin complex can help control bleeding [9, 14]. The main treatment of AHA focuses on inhibitor eradication relying on immunosuppressive agents such as corticosteroids, rituximab and cyclophosphamide [15, 16]. In the current case, the patient was treated by low dose rituximab (100 mg/week for 4 weeks) associated with corticosteroids. This regimen is preferred than the standard dose (375 mg/m^2^) to limit occurrence of side effects, mainly neutropenia, potentially dangerous to the elderly who have increased sensibility to infections [17].

## Conclusion

This article further highlights potential safety concerns regarding COVID-19 vaccination. Although benefits acquired from vaccination highly overweighs the risks, post-vaccine surveillance is important to detect potential uncommon side-effects. Clinicians should consider AHA in front of prolonged a PTT with or without spontaneous bleedings, which, without prompt treatment, could be a life-threatening disease.

## Data Availability

The dataset of the current study is available from the corresponding author upon motivated request.
